# Behaviour-correlated profiles of cerebellar-cerebral functional connectivity observed in independent neurodevelopmental disorder cohorts

**DOI:** 10.1038/s41398-024-02857-4

**Published:** 2024-04-03

**Authors:** Felipe Morgado, Marlee M. Vandewouw, Christopher Hammill, Elizabeth Kelley, Jennifer Crosbie, Russell Schachar, Muhammad Ayub, Robert Nicolson, Stelios Georgiades, Paul Arnold, Alana Iaboni, Azadeh Kushki, Margot J. Taylor, Evdokia Anagnostou, Jason P. Lerch

**Affiliations:** 1 Dept. Medical Biophysics, University of Toronto, Toronto, Canada; 2 Neurosciences & Mental Health, Hospital for Sick Children, Toronto, Canada; 3 Institute of Biomedical Engineering, University of Toronto, Toronto, Canada; 4 Autism Research Centre, Holland Bloorview Kids Rehabilitation Hospital, Toronto, Canada; 5 Data Science & Advanced Analytics, St. Michael’s Hospital, University of Toronto, Toronto, Canada; 6 Department of Psychiatry, Queen’s University, Kingston, Canada; 7 Department of Psychiatry, University of Toronto, Toronto, Canada; 8 Department of Psychiatry, University College London, London, UK; 9 Department of Psychiatry, University of Western Ontario, London, Canada; 10 Lawson Research Institute, London, Canada; 11 Department of Psychiatry and Behavioural Neurosciences, McMaster University, Hamilton, Canada; 12 Offord Centre for Child Studies, McMaster University, Hamilton, Canada; 13 The Mathison Centre for Mental Health Research & Education, Hotchkiss Brain Institute, University of Calgary, Calgary, Canada; 14 Department of Psychiatry, University of Calgary, Calgary, Canada; 15 Diagnostic Imaging, Hospital for Sick Children, Toronto, Canada; 16 Department of Medical Imaging, University of Toronto, Toronto, Canada; 17 Institute of Medical Science, University of Toronto, Toronto, Canada; 18 Nuffield Department of Clinical Neurosciences, Oxford University, Oxford, UK

**Keywords:** Predictive markers, Neuroscience

## Abstract

The cerebellum, through its connectivity with the cerebral cortex, plays an integral role in regulating cognitive and affective processes, and its dysregulation can result in neurodevelopmental disorder (NDD)-related behavioural deficits. Identifying cerebellar-cerebral functional connectivity (FC) profiles in children with NDDs can provide insight into common connectivity profiles and their correlation to NDD-related behaviours. 479 participants from the Province of Ontario Neurodevelopmental Disorders (POND) network (typically developing = 93, Autism Spectrum Disorder = 172, Attention Deficit/Hyperactivity Disorder = 161, Obsessive-Compulsive Disorder = 53, mean age = 12.2) underwent resting-state functional magnetic resonance imaging and behaviour testing (Social Communication Questionnaire, Toronto Obsessive-Compulsive Scale, and Child Behaviour Checklist – Attentional Problems Subscale). FC components maximally correlated to behaviour were identified using canonical correlation analysis. Results were then validated by repeating the investigation in 556 participants from an independent NDD cohort provided from a separate consortium (Healthy Brain Network (HBN)). Replication of canonical components was quantified by correlating the feature vectors between the two cohorts. The two cerebellar-cerebral FC components that replicated to the greatest extent were correlated to, respectively, obsessive-compulsive behaviour (behaviour feature vectors, *r*_*POND-HBN*_ = −0.97; FC feature vectors, *r*_*POND-HBN*_ = −0.68) and social communication deficit contrasted against attention deficit behaviour (behaviour feature vectors, *r*_*POND-HBN*_ = −0.99; FC feature vectors, *r*_*POND-HBN*_ = −0.78). The statistically stable (|*z*| > 1.96) features of the FC feature vectors, measured via bootstrap re-sampling, predominantly comprised of correlations between cerebellar attentional and control network regions and cerebral attentional, default mode, and control network regions. In both cohorts, spectral clustering on FC loading values resulted in subject clusters mixed across diagnostic categories, but no cluster was significantly enriched for any given diagnosis as measured via chi-squared test (*p* > 0.05). Overall, two behaviour-correlated components of cerebellar-cerebral functional connectivity were observed in two independent cohorts. This suggests the existence of generalizable cerebellar network differences that span across NDD diagnostic boundaries.

## Introduction

The cerebellum, while classically viewed as a structure exclusively linked to motor control, is now understood to have a much broader scope of activity. Roughly 30 years of research have contributed strong evidence supporting that the cerebellum plays an integral role in the regulation of cognitive and affective processes, largely through its communication between its posterior lobe and higher-order and association cerebral cortices [1–3]. Structural [4, 5] and functional imaging [6, 7] in humans, as well as tract-tracing studies in preclinical models [8], have contributed to this evidence.

Studies have shown that dysregulation of cerebellar pathways implicated in cognitive and affective processing can lead to behavioural differences in these domains. In mice, Kelly et al. [9] demonstrated that chemogenetic inhibition of right crus I and the posterior vermis led to disinhibition of the medial prefrontal cortex (under normal function, the cerebellar cortex provides inhibitory regulation of downstream cerebral targets via its GABAergic Purkinje cells). These disinhibited mice were observed to have attenuated social behaviour in multiple rodent behaviour tests [9]. In humans, dysfunction of the posterior cerebellum due to injury or reduced blood supply has also been associated with reduced social and cognitive function. In a study of perinatal cerebellar injury in premature infants, Limperopoulos et al. observed cognitive deficits, language deficits, and externalizing behaviours issues at 2 years of age in nearly half of the infants with cerebellar injury [10]. Given that the cerebellum undergoes rapid development in the first two postnatal years of an infant’s life [11], it is conceivable that dysfunction in this region during a period of major growth would lead to persistent functional impairment.

Such studies have spurred further investigation into the potential relationship between changes in cerebellar functional connectivity (FC) and neurodevelopmental disorders (NDDs), and cognitive and affective differences observed with cerebellar dysfunction. For instance, meta-analyses by D’Mello and Stoodley have elucidated convergent volumetric and FC findings that consistently associate cerebellar changes with certain behaviours related to autism spectrum disorder (ASD) in autistic individuals [1]. It has also been shown that perinatal cerebellar injury carries the greatest relative likelihood for ASD development of any noninheritable factor [12]. ASD falls within the broader spectrum of NDDs, encompassing conditions such as attention-deficit/hyperactivity disorder (ADHD) and obsessive-compulsive disorder (OCD). Investigations involving individuals with these conditions have also demonstrated an association between cerebellar atypicalities and relevant behavioural differences [13–17]. Increasingly, researchers, spurred by institutions such as the National Institute for Mental Health [18] and projects such as PRISM [19], have investigated brain-behaviour relations across clinical diagnoses for NDDs rather than within them due to emerging evidence demonstrating a lack of distinctive aetiology, set of behaviours, or biology that separates these disorders [20–26].

Given the demonstrated role of the cerebellum in regulating cognitive and affective behaviours, investigating differences in FC of the cerebellum and its main downstream target, the cerebral cortex, across a range of NDDs may provide a richer characterization of how differences in cerebellar-cerebral FC relate to behaviour/cognition. FC can be indirectly probed using resting-state functional magnetic resonance imaging (rs-fMRI), which uses blood oxygen level-dependent signal as a proxy measurement of underlying spontaneous neural activity [27, 28]. Findings may then be validated by repeating the investigation in an independent cohort, comprised of subjects recruited by a different consortium.

The aim of this study was therefore to investigate whether discernable patterns of cerebellar-cerebral FC as defined by their relationship to behaviour can be identified among NDD children, in two independent cohorts.

## Methods

### Participants (original cohort)

Participants were recruited through the Province of Ontario Neurodevelopmental Disorders (POND) Network. The POND Network spans four institutions within Ontario, Canada: The Hospital for Sick Children, Toronto; Holland Bloorview Kids Rehabilitation Hospital, Toronto; McMaster Children’s Hospital, Hamilton; Queen’s University, Kingston; and Lawson Health Research Institute, London. The research and data collection protocol were developed by the POND Executive Committee and approved by the Research Ethics Boards at each site. Recruitment of TD participants (i.e., participants with no neurodevelopmental, neurological, or psychiatric diagnosis or first-degree family history thereof, and born after 35 weeks gestation) was promoted via advertisements in hospitals, on social media, and in public transit.

Included participants had no contraindications for MRI, were not included in intervention arms of clinical investigations stemming from the POND Network and possessed a sufficient degree of English comprehension to follow instructions in testing protocols and provide informed consent. The participants included in this study required a primary diagnosis of ASD, ADHD, or OCD or typically developing (TD). Standardized behavioural assessments verified clinical diagnosis using established metrics: Autism Diagnostic Observation Schedule-2 [29] and Autism Diagnostic Interview-Revised [30] for ASD; Parent Interview for Child Symptoms [31] for ADHD; and the Kiddie-Schedule for Affective Disorders and Schizophrenia [32] and the Children’s Yale-Brown Obsessive Compulsive Scale [33] for OCD.

747 participants from the POND Network received rs-fMRI scans between 2010 and 2020. Of those, 603 scans survived quality control (QC) filtering (described below) and were not included in intervention arms of studies. Participants were then excluded if they did not possess any of the following behavioural questionnaire scores: Child Behaviour Checklist [34] Attentional Problems subscore (CBCL), Social Communication Questionnaire [35] (SCQ) total score, and Toronto Obsessive Compulsive Scale [36] (TOCS) total score. These scores were selected because they probe core behavioural symptoms associated with ASD, ADHD, and OCD, and were minimally collinear (partial correlation between any pair of the three scores was less than 0.3) [37]. Weschler Intelligence Scale for Children IQ was also examined [38]. Following exclusion based on missing behaviour scores, 479 participants remained (Table 1).

**Table 1:** Demographics and clinical characteristics of study participants.

	POND	HBN	*P*-value
*N*	479	556	
Age (mean (SD))	12.15 (3.16)	10.81 (3.04)	<0.001
Diagnosis (%)			<0.001
TD	93 (19.4)	113 (20.3)	
ADHD	161 (33.6)	374 (67.3)	
ASD	172 (35.9)	61 (11.0)	
OCD	53 (11.1)	8 (1.4)	
Sex = Male (%)	345 (72.0)	371 (66.7)	0.076
Child Behaviour Checklist - Attentional Problems Subscore (mean (SD))	63.46 (10.44)	63.18 (10.15)	0.66
Social Communication Questionnaire - Total Score (mean (SD))	10.30 (9.09)	7.53 (5.06)	<0.001
Toronto Obsessive-Compulsive Scale - Total Score (mean (SD))	−16.97 (29.07)	N/A	
Child Behaviour Checklist - Obsessive-Compulsive Subscore (mean (SD))	N/A	49.51 (9.82)	
Weschler Intelligence Scale for Children IQ (mean (SD))	102.88 (17.27)	100.72 (16.89)	0.073

### Participants (replication cohort)

Data from the Healthy Brain Network (HBN) were used as an independent replication cohort [39]. HBN is an ongoing initiative to amass behavioural, cognitive and lifestyle phenotypic data, genetic data, and data from structural and functional imaging modalities from 10,000 New York-city area children aged 5 to 21. Participants were recruited from the community using advertisements targeting families with concerns about NDD-related symptoms in their child. Inclusion and exclusion criteria were described in Alexander et al. [39].

Imaging data from 953 HBN participants were downloaded and pre-processed identically to the POND data (described below). 556 of those participants survived QC filtering and had complete records for the CBCL and SCQ. TOCS was not administered to the HBN cohort; thus, the Obsessive-Compulsive subscore from CBCL [40] (CBCL-OCS) was used in its place. In a community sample of 16,718 subjects, CBCL-OCS was observed to correlate with TOCS total score with a Spearman correlation of 0.51 [36].

### Imaging protocol (original cohort)

All rs-fMRI data were collected between June 2012 and September 2020. 160 of 479 (33.4%) scans took place on the 3-Tesla Siemens Trio TIM with a 12-channel head coil, while the remaining 319 (66.6%) scans took place on the 3-Tesla Siemens Prisma scanner with a 20-channel head and neck coil, following a hardware upgrade in 2016 (scanner upgrade occurred at the Queen’s site in 2019). Structural and functional imaging were performed. The parameters for the T1-weighted images were as follows: for Trio, TR/TE = 2300/2.96 ms, FA = 9^o^, FOV = 192 × 240 × 256 mm, 1.0 mm isotropic voxels; for Prisma, TR/TE = 1870/3.14 ms, FA = 9^o^, FOV = 192 × 240 × 256 mm, 0.8 mm isotropic voxels. Scan duration was 5 minutes for both scanners. The parameters for the rs-fMRI scans were as follows: for Trio, TR/TE = 2340/30 ms, FA = 70^o^, FOV = 224 × 224 × 140 mm, 3.5 mm isotropic voxels; for Prisma, TR/TE = 1500/30 ms, FA = 70^o^, FOV = 222 × 222 × 150 mm, 3.0 mm isotropic voxels. Scan duration was 5 min for both scanners. During the resting-state scans, participants scanned in the Trio scanner viewed a movie of their choice during scanning, while participants scanned in the Prisma viewed the Inscapes naturalistic movie paradigm [41]. It has been shown that while movie and Inscape viewing conditions result in reduced motion and fewer participants who fall asleep mid-scan relative to a static image (e.g. fixation cross), inter- and intra-network connectivity metrics may be influenced by viewing condition [41, 42]. Therefore, viewing condition was incorporated as a covariate in the analysis.

### Imaging protocol (replication cohort)

Scans were collected using a Siemens Trio TIM or Siemens Prisma scanner at three sites: Rutgers University Brain Imaging Center (3-Tesla Trio), CitiGroup Cornell Brain Imaging Center (3-Tesla Prisma), and the HBN Diagnostic Research Center in Staten Island, New York (1.5-Tesla Trio). The protocols can be found in Alexander et al. [39] and on the HBN Updates page (http://fcon_1000.projects.nitrc.org/indi/cmi_healthy_brain_network/Updates.html). Participants viewed a fixation-cross while undergoing scanning [39].

### Image pre-processing

Pre-processing and segmentation were performed identically on the replication cohort data from HBN. Results included in this manuscript come from pre-processing performed using fMRIPrep (RRID:SCR_016216) [43], a Nipype-based tool [44]. For detailed methods, see Supplemental Methods. After fMRIPrep, Analysis of Functional NeuroImages (AFNI) was used to perform simultaneous nuisance signal regression, volume censoring, and high-pass temporal filtering [45]. In particular, the 36-parameter (six motion parameters, WM, CSF, and global signal, along with their derivative and quadratic terms) model was used [46], with sinc/cosine bases for high-pass filtering (>0.008 Hz) and censoring volumes with a framewise displacement exceeding 0.5 mm or DVARS exceeding 0.5%.

Quality control was performed following pre-processing. For each scan, volumes were censored if framewise displacement was greater than 0.5 mm or DVARS > 0.5% [47]. Scans were excluded if more than 1/3 of volumes were censored. The remaining scans were visually inspected to assess whether cerebellar coverage of BOLD signal was acceptable; in other words, if there were no areas of missing signal (when observed, this typically occurred at the caudal cerebellum and was likely due to the subject’s placement in the scanner). Scans missing BOLD signal in parts of the cerebellum were also excluded.

### Segmentation

**Fig. 1:**
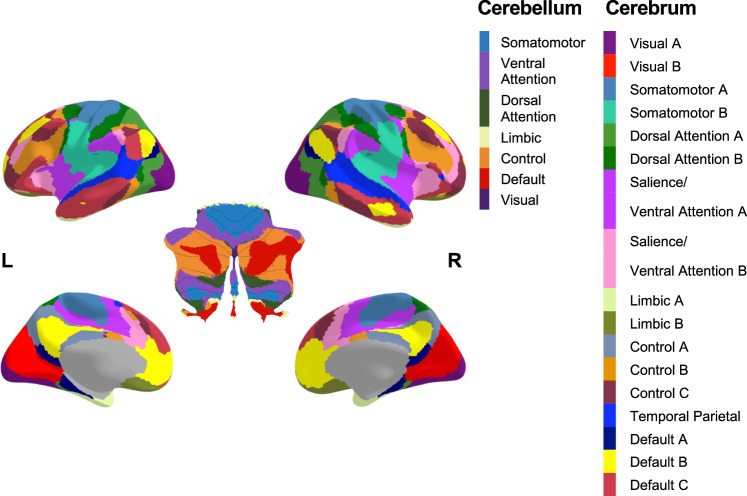
Functionally-derived cerebrum [48] and cerebellum [49] parcellation used in this study. Cerebellar parcellation was overlaid over a flat-map [95]. The control network region of the cerebellar parcellation was originally labelled as “Frontoparietal Network” in Buckner et al. but was renamed in this study to correspond with the region labels of the Yeo et al. cerebral parcellation.

To measure FC, a parcellation to define regional signal was required. The applied parcellation was the combination of complementary studies by Yeo et al. and Buckner et al. (Fig. 1) which derived atlases of the cerebellum and cerebral cortex based on resting-state FC in healthy brains from the Human Connectome Project [48, 49]. The Yeo-Buckner atlas used in this study was comprised of the 7-region-of-interest (ROI) parcellation from the cerebellum combined with the 17-ROI parcellation from the cerebral cortex. Since the ROIs were bilateral, different labels were assigned to left and right-hemispheric sections of regions, resulting in a total of 48 ROIs. The 17-ROI parcellation was used for the cerebral cortex to provide finer spatial resolution. The 7-ROI parcellation was used for the cerebellum because the 17-ROI parcellation failed to register in a consistent manner across all subjects (some small midline ROIs were erased or reduced to a few voxels in roughly one-third of all subjects).

Cerebellar ROIs were segmented using multiple automatically generated templates (MAGeT) [50], with 23 randomly selected scans used as templates. Cerebral cortical structures were segmented using surface registration using the CIVET pipeline (version 2.1.0) [51, 52], which registers scans into a common space, applies corrections for radiofrequency inhomogeneity artifacts [53, 54] and labels cortical regions as either grey matter, white matter, or cerebrospinal fluid [52].

### Functional connectivity

The measurement of functional connectivity across participants and the subsequent statistical analyses were conducted using the programming language R (v 3.5.1). Code is available upon request. For each resting-state scan, ROI-level BOLD signal was defined as the mean time series of the voxels within each ROI. Pearson’s correlation was calculated between the time series of each pair of ROIs to generate a correlation matrix for each scan. Correlation matrices were then transformed to partial correlation matrices, which can be calculated using the following equation [55]:

1 $${\Pi }_{\left\{{ij}\right\}}=-\frac{{{\rm{{\Upsilon }}}}_{\left\{{ij}\right\}}}{\sqrt{{{\rm{{\Upsilon }}}}_{\left\{{ii}\right\}}{{\rm{{\Upsilon }}}}_{\left\{{jj}\right\}}}}$$

for distinct *i* and *j* (${\Pi }_{\left\{{ii}\right\}}=1$). $\Pi$ is the partial correlation matrix, and ${\rm{{\Upsilon }}}={\Sigma }^{-1}$, where $\Sigma$ is the Pearson correlation matrix. Partial correlation represents the correlation between two signals in a subspace that is orthogonal to all other signals; hence, it is a measurement of the correlation between two signals that are independent of the influence of other signals in the network [56]. It has been shown that, compared to full correlation measurements (i.e., Pearson correlation), partial correlations have greater accuracy in identifying connections in networks simulated from dynamic causal modelling principles [57], and have greater reproducibility for various network-wide measures when tested on real data [58]. The correlation data were controlled for differences between scan sites using the ComBat empirical Bayes package in R [59]. Sex and viewing condition were controlled by linearly regressing out their effects on the correlation data. Age was not regressed out in order to later test for age differences between clusters of subjects—see Spectral Clustering subsection for details. The resulting residuals were used in this study as our measure of functional connectivity (FC).

### Canonical correlation analysis

The aim of this work was to investigate the existence of stable and discriminable cerebellar-cerebral FC patterns of expression that presented with specific behavioural profiles in children with NDDs. Thus, we sought a maximally correlated transformation of the FC and behavioural data, which can be achieved using canonical correlation analysis (CCA) [60, 61]. The FC data consisted of the Fisher-transformed [62] pairwise partial correlations between ROIs (i.e., FC features), while the behavioural data consisted of the centred and normalized questionnaire scores. CCA was performed in R using the packages CCA (v 1.2.1) and CCP (v 1.1) [63].

Similar to Drysdale et al. [64], CCA was performed using only FC features that correlated with behaviour (Spearman’s correlation) with a *t*-test-derived *p*-value less than 0.05. This step was performed to improve the correlation between FC and behaviour by eliminating weakly-correlating FC features. To assess the effect of applying a more stringent FC-behaviour threshold on the results, the analysis was repeated at a different threshold choice (*p* < 0.01). Behaviour and FC coefficients were largely similar regardless of threshold choice (Supplementary Fig. 1).

Given the cerebellum’s major role in coordinating motor function in response to sensorimotor feedback, a supplemental analysis was performed to characterize the correlation between cerebellar-cerebral FC and sensory-related behaviours. Short Sensory Profile [65] (SSP) total score was included among the examined behaviour scores and CCA was re-run. Replication was not assessed for this supplemental analysis given the absence of SSP or a related measure of the same domain among the behaviour tests administered to the HBN participants.

Statistical significance of canonical variates extracted from CCA was measured using a permutation test approach with 10,000 shuffled samples. Statistical stability of canonical coefficients was assessed by bootstrap sampling the data, re-running CCA 10,000 times, storing the canonical coefficients from all canonical variates after each iteration, measuring the standard deviation of each coefficient over all iterations, then dividing the original non-bootstrap coefficients by the bootstrap-measured standard deviation. This resulted in a *z*-score for each coefficient. Larger *z* denoted a more stable coefficient, and |z| > 1.96 was used as the threshold for statistical stability [60, 61]. Procrustes transformations were applied with each re-sampling to account for rotations in the data relative to the original data matrices [60].

### Spectral clustering

To investigate whether diagnosis-specific cerebellar-cerebral FC profiles were observable across NDD and TD subjects, spectral clustering was performed on the statistically significant canonical variates (CVs) of the FC data using the SNFtool package in R [66]. The affinity matrix *A* was calculated by applying a weighting kernel (kernel dampening coefficient $\mu =0.3$, neighbourhood width = 10) to the matrix of subject FC CV loading values. Clustering was then performed on the Laplacian matrix $L=D-A$, where *D* is the diagonal matrix ${D}_{\left\{{ii}\right\}}=\sum _{j}{A}_{\left\{{ij}\right\}}$ [66]. The first *k* eigenvectors of *L* were used to cluster the data using the k-means algorithm [67, 68]. A range of k from 2 to 10 were investigated.

Clustering discriminability was assessed by plotting the Calinhara-Harabasz index as a function of number of clusters [69]. Cluster stability was assessed by measuring the distribution of the Rand Index and adjusted Rand Index [70] between the original clustering and clustering following bootstrap sampling without replacement for 10,000 resamples (adjusted Rand Index corrects for the number of expected agreements by chance for a given number of clusters). Following selecting the optimal number of clusters for the data, the inter-cluster differences in age, IQ, and in-scanner motion (measured as framewise displacement) were assessed using ANOVAs, and in diagnostic category counts using Pearson’s chi-squared test.

### Replication cohort analysis

The measurement of FC and the ensuing CCA and spectral clustering were performed identically in the replication cohort, except for CBCL-OCS being used to assess obsessive-compulsive behaviour severity rather than TOCS total score. To evaluate the similarity between CVs from the original POND cohort and the replication HBN cohort, Pearson’s correlation was measured between the canonical coefficient vectors of the POND and HBN datasets. Behaviour vectors consisted of the coefficients for CBCL Attentional Problems subscore, SCQ total score, and TOCS total score for the POND CVs, and CBCL Attentional Problems subscore, SCQ total score, and CBCL-OCS for the HBN CVs. FC vectors consisted of stable features common to both sets of CVs (FC features calculated from the POND and HBN cohorts were different due to the thresholding step implemented prior to CCA [64]. Prior to correlating between datasets, FC coefficients were recalculated over a parcellation in which subdivisions of the same network were combined into a single bilateral region (e.g., combining anterior and posterior default mode network regions from the left and right brain into one default mode region). Replication was achieved if a CV from the POND cohort strongly correlated to a CV from the HBN cohort, with respect to their behaviour coefficient vectors and their FC coefficient vectors (e.g. POND CV 1’s behaviour and FC coefficient vectors strongly correlated to HBN CV 2’s behaviour and FC coefficient vectors).

## Results

### Canonical variates—original cohort

**Fig. 2:**
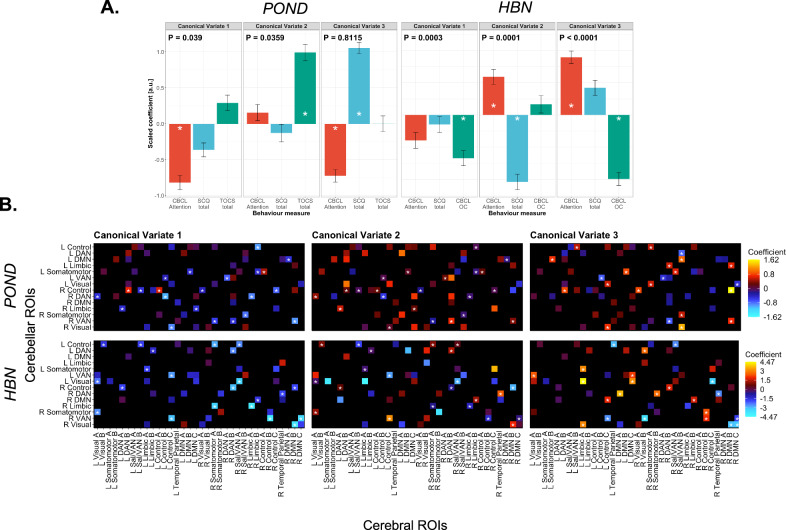
Canonical correlation analysis on POND and HBN cohort data. **A** Standardized canonical coefficients for behaviour scores measured from the original (i.e., POND) and replication (i.e., HBN) cohort. Errors bars indicate the standard deviation of canonical coefficients recalculated over 10,000 bootstrap resamples. * denotes |z| > 1.96, where z is the ratio of a coefficient to its standard error. If |z| > 1.96, the coefficient is considered to be stable. **B** Canonical coefficients for functional connectivity features. * denotes stable coefficients. The top row pertains to the original cohort and the bottom row pertains to the replication cohort. CBCL Child Behaviour Checklist, SCQ Social Communication Questionnaire, TOCS Toronto Obsessive-Compulsive Scale, DAN dorsal attention network, VAN ventral attention network, DMN default mode network, Sal salience network.

Two statistically significant and one non-significant CV were identified (CV 1, *p* = 0.0390; CV 2, *p* = 0.0359; CV 3, *p* = 0.811) (Fig. 2A). CV 1 and CV 2 were most weighted for one behavioural score over the other two, as indicated by the relative size of the canonical coefficients and the observed statistical stability (CBCL Attentional Problems subscore for CV 1 and TOCS total score for CV 2—see Methods—‘Canonical correlation analysis’ for how canonical coefficient stability was defined). CV 3 was characterized by two stable coefficients of opposite sign, with the absolute value of the SCQ total score coefficient being greater than the CBCL Attentional Problems subscore coefficient (Supplementary Table 1, Supplementary Fig. 2). The 3 CVs were subsequently referred to as the Attention Deficit CV, the Obsessive-Compulsive CV, and the Social Communication Deficit contrasted to Attention Deficit (“Social-versus-Attention”) CV. The canonical coefficients are interpreted as the amount by which a given measure changes given a 1 unit change in its paired canonical variate. For example: a 1 unit increase in the first CV of the functional connectivity data was associated with an 0.81 unit decrease in the normalized CBCL Attentional Problems subscore in the first CV of the behaviour data.

The FC profiles for each CV can be characterized by their observed stable coefficients (Fig. 2B). The Attention Deficit CV and Obsessive-Compulsive CV were generally characterized by stable FC features between cerebellar attentional and control network regions and cerebral dorsal attention, default mode, and control network regions. Stable FC was also observed for features involving limbic network regions, somatomotor network regions (predominantly cerebellar somatomotor region), and visual network regions (predominantly cerebellar visual network region for the Obsessive-Compulsive CV). The Social-versus-Attention CV was characterized by stable FC mostly between cerebellar control, visual, somatomotor, and attentional network regions and cerebral default mode and attentional network regions. No patterns suggesting any lateralization among the stable FC features were observed (Supplementary Table 2). Most stable coefficients shared the same sign as their respective stable behaviour score. In the case of the Social-versus-Attention-CV, this was true for the stable behaviour score with the largest absolute coefficient (SCQ total score). This indicated a positive correlation between FC feature partial correlation and phenotypic severity for most of the stable FC features.

### Sensory-related behaviour analysis—original cohort

**Fig. 3:**
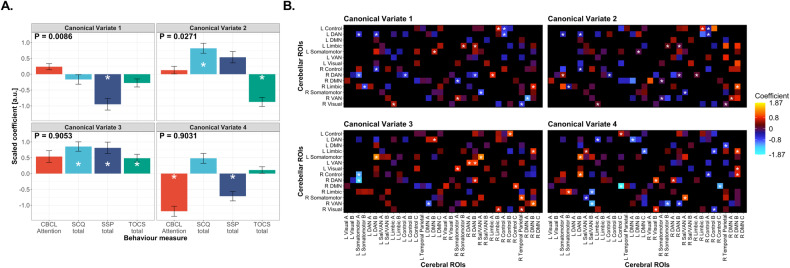
Canonical correlation analysis on POND data with Short Sensory Profile score added. **A** Standardized canonical coefficients for behaviour scores measured from the original (i.e., POND) cohort. Short Sensory Profile total score was added to the set of analysed behaviour scores. Errors bars indicate the standard deviation of canonical coefficients recalculated over 10,000 bootstrap resamples. * denotes |z| > 1.96, where z is the ratio of a coefficient to its standard error. If |z| > 1.96, the coefficient is considered to be stable. **B** Canonical coefficients for functional connectivity features after including SSP total score. * denotes stable coefficients. CBCL Child Behaviour Checklist, SCQ Social Communication Questionnaire, TOCS Toronto Obsessive-Compulsive Scale, DAN dorsal attention network, VAN ventral attention network, DMN default mode network, Sal salience network.

Incorporating SSP total score into the analysis demonstrated that sensory-related behaviour correlated with functional connectivity involving the cerebellar and cerebral somatomotor network regions. Only the first CV of four was statistically significant on permutation testing (CV 1, *p* = 8.60 × 10^−3^) and bore a stable coefficient for SSP total score (Fig. 3). Two other CVs also bore stable SSP total score coefficients but were not statistically significant (CV 3, *p* = 0.905; CV 4, *p* = 0.903). The three CVs with stable coefficients for SSP total score (CV 1, 3, and 4) had a greater proportion of stable FC features involving the somatomotor cerebellar or cerebral network regions than CV 2 (CV 1, 38.1% of stable features; CV 2, 13.3%; CV 3, 35.3%; CV 4, 19.2%).

### Clustering—original cohort

**Fig. 4:**
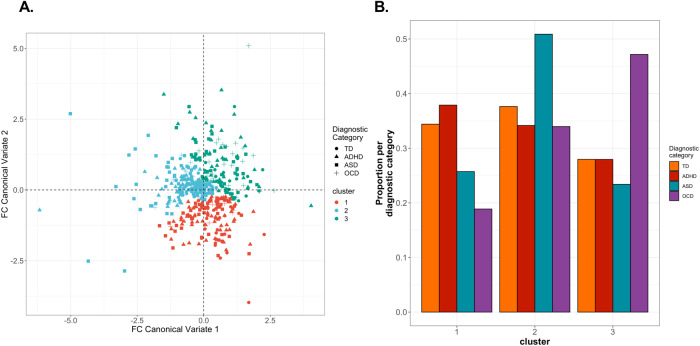
Clustering POND cohort subjects according to functional connectivity. **A** Subject cluster assignment following spectral clustering on functional connectivity canonical variate loading values. Only canonical variates 1 and 2 were used since canonical variate 3 did reach statistical significance on permutation testing. **B** Proportion of subjects per diagnostic category per cluster.

Clusters of participants based on behaviour-correlated cerebellar-cerebral FC were not distinct, and no diagnosis-specific cluster was observed. Participants were clustered using their loading values for the Attention Deficit and Obsessive-Compulsive FC CVs since these components were significant on permutation testing (Fig. 4). The three-cluster solution resulted in the largest Calinhara-Harabasz index value and median adjusted Rand index value (Supplementary Fig. 3). Participants across all four diagnostic categories were represented in each cluster. Across the three clusters, the proportion of participants per diagnostic category was not significantly different from the proportion across the whole POND cohort (cluster one, X^2^(df) = 5.40 (3), *p* = 0.15; cluster 2, X^2^(df) = 4.74 (3), *p* = 0.19; cluster three, X^2^(df) = 5.70 (3), *p* = 0.13). Clusters were not well-isolated in FC CV space, suggesting weakness in the evidence towards a clustering structure in behaviour-correlated cerebellar-cerebral FC in NDDs. The three clusters were not significantly different on in-scanner motion or age, as measured by ANOVAs (Supplementary Table 3), but were significantly different with respect to IQ (F_2,291_ = 3.53, *p* = 0.03). However, the difference in median IQ between clusters was small (106, 101, 106) (Supplementary Fig. 4).

### Replication cohort—canonical variates

All three identified CVs in the replication dataset were significant (CV 1, *p* = 3.00 × 10^−4^; CV 2, *p* = 1.00 × 10^−4^; CV 3, *p* < 10^-4^) (Fig. 2, Supplementary Fig. 5). CV 1 was characterized by a stable coefficient for CBCL Obsessive-Compulsive subscore, CV 2 was characterized by stable coefficients of opposite sign for SCQ total score and CBCL Attentional problems Subscore (SCQ total score bore the larger absolute coefficient), and CV 3 was characterized by stable coefficients of opposite sign for CBCL Obsessive-Compulsive subscore and CBCL Attentional Problems subscore, with the Obsessive-Compulsive subscore having the larger absolute coefficient (Supplementary Table 4). Stable FC features across all CVs were generally comprised of visual, attentional, limbic, control, and somatomotor network cerebellar regions and control, default mode, attentional, and somatomotor network cerebral regions.

### Canonical variate replication analysis

**Fig. 5:**
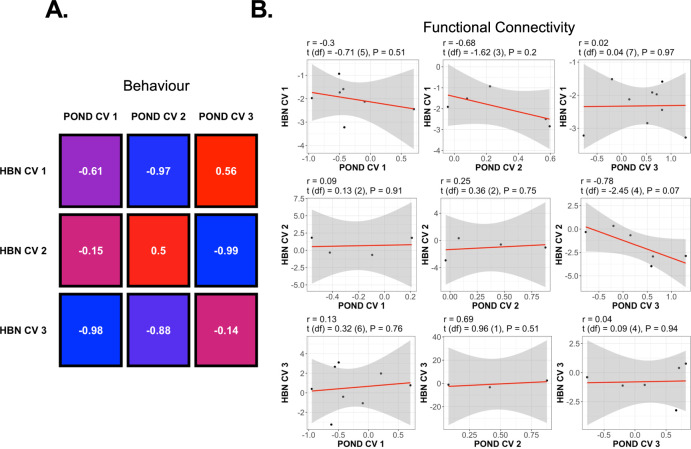
Results of canonical variate replication analysis. **A** Pearson’s correlation between behaviour score canonical variates measured in the POND and HBN cohorts. **B** Pearson’s correlation between common stable features for each CV pair.

The Social-versus-Attention CV from the original (i.e., POND) cohort replicated to the greatest extent in the replication (i.e., HBN) cohort, followed by the Obsessive-Compulsive CV. The Attention Deficit CV did not replicate (Fig. 5). The Social-versus-Attention CV (POND CV 3) correlated most strongly with HBN CV 2 with respect to their vectors of behaviour coefficients (*r* = −0.99, t(df) = 7.02 (1), *p* = 0.03 for a two-tailed test), and their vectors of stable FC coefficients (*r* = −0.78, t(df) = −2.45 (4), *p* = 0.07). Although the Attention Deficit CV (POND CV 1) strongly correlated with HBN CV 3 in terms of behaviour coefficients (*r* = −0.98, t(df) = 4.92 (1), *p* = 0.04), the correlation between FC coefficients was weak (*r* = 0.13, t(df) = 0.32 (6), *p* = 0.76). Shared FC features observed in both original and replication cohorts most often included the cerebellar attentional and control network regions, and the cerebral default mode, control and ventral attention network regions (Supplementary Table 5). To a lesser extent, shared FC features also included the somatomotor and visual network regions. Note that the negative sign of correlations between POND and HBN coefficient vectors is a product of feature space rotation due to the canonical correlation analysis process [61] rather than a true anticorrelation between the two sets of results. The general relationship between the signs of the behaviour and FC coefficients for each canonical coefficient was preserved. For example, most stable FC coefficients in POND CV 3 were of the same sign as the SCQ total score coefficient; this was similarly true for HBN CV 2, which was highly correlated to POND CV 3.

### Clustering—replication cohort

Applying spectral clustering with a three-cluster solution on the subject loading values from the significant FC CVs (all three CVs in the case of the replication cohort) resulted in clusters that were well-mixed across diagnostic categories, as observed in the POND cohort (Supplementary Fig. 6). Chi-squared testing revealed no significant difference in the relative frequency of participants per diagnostic category per cluster compared to the frequencies across the whole sample. ANOVA testing showed no significant difference in age, IQ, or in-scanner motion between subjects across the three clusters (Supplementary Table 3).

## Discussion

In two independent subject cohorts from different institutional networks, behaviour-correlated components of cerebellar-cerebral FC were identified across NDD children, characterizing patterns of co-activity between the cerebellum and its main downstream target for non-motor behaviour regulation, the cerebrum. Strikingly, two of three identified components replicated when analysis was repeated in the replication dataset. The components observed in both the original and replication cohort were a social communication difference-versus attention deficit component and an obsessive-compulsive component. The former component, despite being non-significant in the original cohort, constituted a stronger replication as evidenced by the similar behaviour coefficient sizes and the high correlation to stable FC feature coefficients to component from the replication dataset. FC features shared among the CVs measured in both cohorts mainly included the cerebellar attentional and control network regions and the cerebral default mode, control, and ventral attention network regions.

To our knowledge, no other study has investigated cerebellar-cerebral FC differences across multiple NDDs. To make sense of the findings with respect to the literature, we therefore compared to diagnosis-specific investigations most relevant to the maximally correlated behaviours of each CV. For the Attention Deficit and Obsessive-Compulsive CVs measured in the POND cohort (CVs 1 and 2), stable FC mainly included the attentional and control network regions of the cerebellum and the dorsal attention, default mode and control network regions of the cerebrum. Atypical connectivity involving all listed cerebral regions have been observed in ADHD relative to TD [71–74]. Attentional network abnormalities are a common finding in individuals with ADHD [75–77], as it was in the Attention Deficit CV (POND CV 1), and disruptions are believed to influence activity in other networks, especially the default mode network [71]. With respect to OCD FC, deficits in switching between task-positive and default mode network activation in the cerebrum during cognitive tasks are considered to be a hallmark [78, 79] (disruptions to these networks have also been observed in resting-state FC [80, 81]). The contribution of the attentional, control, and default mode networks to the stable features of the FC CVs evokes the triple network model of control network-mediated switching between salient and non-salient states [82, 83], which motivates further investigation into cerebellum-mediated triple network activity among children with NDDs.

The presence of a CV with stable social communication deficit and attention deficit features (i.e., the Social-versus-Attention CV or POND CV 3)—as opposed to a solely defined social communication deficit CV—may be due to a couple factors. Of the behaviour scores investigated, SCQ total score and CBCL Attentional Problems subscore were the two most strongly correlated (*r* = 0.42 over all POND participants, compared to *r* = 0.17 for CBCL Attentional Problems subscore and TOCS total score and *r* = 0.25 for SCQ total score and TOCS total score). This was not surprising as attention deficits are common among children with ASD [21, 22]. Secondly, the greater prevalence of stable somatomotor and visual network features in the Social-versus-Attention CV compared to the Attention Deficit CV may reflect the theorized causal relationship between early-life sensory behaviour disruptions and the later manifestation of social communication and attention deficits. This is referred to as the developmental cascade hypothesis [84, 85].

Given the major role the cerebellum plays in sensory feedback-informed coordination, this relationship between these behaviours was expected to correlate with cerebellar-cerebral FC disruption. This was highlighted in the supplemental analysis involving Short Sensory Profile behaviour scores in POND participants, which demonstrated greater somatomotor network involvement in CVs characterized by sensory-related behaviour deficits (Fig. 3). This analysis also reflected previous studies such as Khan et al. which also showed greater connectivity between sensory and motor networks and non-motor networks in ASD [86]. Further investigation is warranted to probe the relationship between cerebellar-cerebral FC and autism-related behaviours in the context of specific sensory-related behaviour deficits, rather than the composite score used here comprised of multiple subscales. Longitudinal studies would also be greatly beneficial to directly investigate the causality implicated by the developmental cascade hypothesis. Such future studies could also help explain or refute why attention deficit severity was anti-correlated with social communication deficit severity in the Social Communication-versus-Attention Deficit CV.

While the Social-versus-Attention-CV possessed features reflective of FC disruptions in ASD and while it replicated in the replication cohort dataset, it was non-significant for the original cohort. Given that the CV was significant in the replication cohort (*p* = 1 × 10^−4^), this may be attributable to the smaller sample size of the original cohort compared to the replication cohort. Differences in behaviour score distributions between the cohorts may have also been a factor, as POND SCQ Total Scores possessed a right-tailed skew in their distribution due to few subjects with severe social communication deficits.

No diagnosis-specific or diagnosis-enriched subgroup was observed after clustering subjects based on cerebellar-cerebral FC. In both the original and replication cohort, clustered subjects were mixed across diagnoses, with no significant difference in age or in-scanner motion (a significant difference in IQ was measured between clusters in the original cohort, but the median IQ range spanned only 5 points). Such an overlap in behaviour phenotype and brain endophenotype across diagnoses has been repeatedly observed [21, 23, 87]. This supports the case for future investigations of NDD cerebellar-cerebral FC to not be restricted to single diagnoses such as ASD but rather consider behaviour and cerebellar function as a continuum that changes along the NDD spectrum. These findings also suggest that cerebellar atypicalities present in children with NDDs are driven by general neurodevelopment differences rather than biological mechanisms specific to any one NDD diagnosis.

It is clear from Table 1 that notable differences exist between the original POND cohort and the replication HBN cohort. The proportion of subjects from each diagnostic category across were notably different (the HBN cohort comprised of proportionately many more ADHD subjects and many fewer OCD subjects (Table 1)). SCQ total score distributions were also dissimilar between cohorts, with the POND cohort possessing proportionally more participants with higher scores (CBCL Attention subscore, on the other hand, was not significantly different). These observations may be attributed to differences in cohort demographics, such as the younger mean age and lesser proportion of males in the HBN versus the POND cohort (Table 1). More importantly, POND and HBN significantly differed in their participant recruitment strategies: POND recruited NDD children through hospital sites and required a primary diagnosis of an NDD, whereas HBN recruited children with through community advertisements by targeting caregivers who suspect that their child meets criteria for an NDD diagnosis. Caregivers of HBN participants were incentivized by providing them with comprehensive diagnostic evaluations, clinical impressions and actionable treatment recommendations which could subsequently be used to acquire an Individual Education Programme for their child. In addition, unlike for POND, HBN exclusion criteria contained an exclusion for children with moderate to severe cognitive impairment (IQ < 66) [39]. Despite these differences, we observed replication of two components, suggesting that common patterns of cerebellar-cerebral FC exist across the heterogeneous NDD population. This study addresses the need for more validation of findings in independent cohort in brain-behaviour investigations [88–90]; this is facilitated by increasing efforts to make consortia data publicly available.

Finally, the Yeo and Buckner et al. parcellations employed here—although widely used and in agreement with functional parcellations derived by alternative methods such as by Guell et al. [91]—represent but one possible functional parcellation of the cerebellum and cerebrum [92–94]. The Yeo and Buckner parcellation with the greatest number of regions (17) was used for the cerebrum and was initially intended to be used for the cerebellum as well. However, the 7-region version was used instead due to inconsistent registration of the 17-region version to the cerebellum across all scans. The number of regions is inversely related to region size, which affects the calculated average BOLD signals (larger regions generally exhibited more heterogenous BOLD signal).

## Conclusion

Cerebellar functional connectivity in children with neurodevelopmental conditions was observed to span two behaviour-correlated components. These components correlated, respectively, to the severity of obsessive-compulsive behaviours and social communication differences contrasted against attention deficit. The most heavily weighted, stable, and replicable features of cerebellar functional connectivity were between cerebellar attentional and control network regions and cerebral attentional, default mode and control network regions. No clear distinction in cerebellar functional connectivity by diagnostic category was observed, suggesting that cerebellar network atypicalities need to be understood transdiagnostically.

### Supplementary information

Supplemental Methods

Supplemental Figures and Tables

## Data Availability

Demographic, medical history data, behavioural and cognitive assessments for children and youth recruited by the Province of Ontario Neurodevelopmental Disorders Network is available at https://www.braincode.ca/content/controlled-data-releases#pond. Imaging and phenotypic data collected by the Healthy Brain Network is available at http://fcon_1000.projects.nitrc.org/indi/cmi_healthy_brain_network/index.html.
