# Supplementary material for: Behaviour-correlated profiles of cerebellar-cerebral functional connectivity observed in independent neurodevelopmental disorder cohorts
Source: Transl Psychiatry. 2024 Apr 3;14:173. doi: 10.1038/s41398-024-02857-4 (PMC10991387; doi:10.1038/s41398-024-02857-4)
Supplement: Supplementary file 1 — Supplemental Methods [file 41398_2024_2857_MOESM1_ESM.docx]

Supplemental imaging methods for “Behaviour-Correlated Profiles of Cerebellar-Cerebral Functional Connectivity Observed In Independent Neurodevelopmental Disorder Cohorts” by Morgado et al.

Supplemental Methods

Each T1w (T1-weighted) volume was corrected for INU (intensity non-uniformity) using N4BiasFieldCorrection v2.1.0^1^ and skull-stripped using antsBrainExtraction.sh v2.1.0 (using the OASIS template). Brain surfaces were reconstructed using recon-all from FreeSurfer v6.0.1^2^ [RRID:SCR_001847], and the brain mask estimated previously was refined with a custom variation of the method to reconcile ANTs-derived and FreeSurfer-derived segmentations of the cortical gray-matter of Mindboggle^3^ [ RRID:SCR_002438]. Spatial normalization to the ICBM 152 Nonlinear Asymmetrical pediatric template (4.5-18.5 years)^4^ [RRID:SCR_008796] was performed through nonlinear registration with the antsRegistration tool of ANTs v2.1.0^5^ [RRID:SCR_004757], using brain-extracted versions of both T1w volume and template. Brain tissue segmentation of cerebrospinal fluid (CSF), white-matter (WM) and gray-matter (GM) was performed on the brain-extracted T1w using fast^6^ (FSL v5.0.9, RRID:SCR_002823).

Functional data was slice time corrected using 3dTshift from AFNI v16.2.07^7^ [RRID:SCR_005927] and motion corrected using mcflirt^5^ (FSL v5.0.9). "Fieldmap-less" distortion correction was performed by co-registering the functional image to the same-subject T1w image with intensity inverted^8,9^ constrained with an average fieldmap template^10^, implemented with antsRegistration (ANTs). This was followed by co-registration to the corresponding T1w using boundary-based registration^11^ with six degrees of freedom, using bbregister (FreeSurfer v6.0.1). Motion correcting transformations, field distortion correcting warp, BOLD-to-T1w transformation and T1w-to-template (MNI) warp were concatenated and applied in a single step using antsApplyTransforms (ANTs v2.1.0) using Lanczos interpolation.

Physiological noise regressors were extracted applying CompCor^12^. Principal components were estimated for the two CompCor variants: temporal (tCompCor) and anatomical (aCompCor). A mask to exclude signal with cortical origin was obtained by eroding the brain mask, ensuring it only contained subcortical structures. Six tCompCor components were then calculated including only the top 5% variable voxels within that subcortical mask. For aCompCor, six components were calculated within the intersection of the subcortical mask and the union of CSF and WM masks calculated in T1w space, after their projection to the native space of each functional run. Frame-wise displacement^13^ was calculated for each functional run using the implementation of Nipype.

Many internal operations of FMRIPREP use Nilearn^14^ [RRID:SCR_001362], principally within the BOLD-processing workflow. For more details of the pipeline see <https://fmriprep.readthedocs.io/en/stable/workflows.html>.

REFERENCES

1. Tustison, N. J., Avants, B. B., Cook, P. A., Zheng, Y., Egan, A., Yushkevich, P. A., *et al.* N4ITK: Improved N3 Bias Correction. *IEEE Trans. Med. Imaging* **29**, 1310–1320 (2010).

2. Dale, A. M., Fischl, B. & Sereno, M. I. Cortical Surface-Based Analysis: I. Segmentation and Surface Reconstruction. *Neuroimage* **9**, 179–194 (1999).

3. Klein, A., Ghosh, S. S., Bao, F. S., Giard, J., Häme, Y., Stavsky, E., *et al.* *Mindboggling Morphometry of Human Brains*. *PLoS Computational Biology* vol. 13 (2017).

4. Fonov, V. S., Evans, A. C., McKinstry, R. C., Almli, C. R. & Collins, D. L. Unbiased nonlinear average age-appropriate brain templates from birth to adulthood. *Neuroimage* **47**, S102 (2009).

5. Jenkinson, M., Bannister, P., Brady, M. & Smith, S. Improved Optimization for the Robust and Accurate Linear Registration and Motion Correction of Brain Images. *Neuroimage* **17**, 825–841 (2002).

6. Zhang, Y., Brady, M. & Smith, S. Segmentation of brain MR images through a hidden Markov random field model and the expectation-maximization algorithm. *IEEE Trans. Med. Imaging* **20**, 45–57 (2001).

7. Cox, R. W. AFNI: Software for Analysis and Visualization of Functional Magnetic Resonance Neuroimages. *Comput. Biomed. Res.* **29**, 162–173 (1996).

8. Huntenberg, J. Evaluating nonlinear coregistration of BOLD EPI and T1w images. (Freie Universitat Berlin, 2014).

9. Wang, S., Peterson, D. J., Gatenby, J. C., Li, W., Grabowski, T. J. & Madhyastha, T. M. Evaluation of field map and nonlinear registration methods for correction of susceptibility artifacts in diffusion MRI. *Front. Neuroinform.* **11**, 1–9 (2017).

10. Treiber, J. M., White, N. S., Steed, T. C., Bartsch, H., Holland, D., Farid, N., *et al.* Characterization and Correction of Geometric Distortions in 814 Diffusion Weighted Images. *PLoS One* **11**, e0152472 (2016).

11. Greve, D. N. & Fischl, B. Accurate and robust brain image alignment using boundary-based registration. *Neuroimage* **48**, 63–72 (2009).

12. Behzadi, Y., Restom, K., Liau, J. & Liu, T. T. A component based noise correction method (CompCor) for BOLD and perfusion based fMRI. *Neuroimage* **37**, 90–101 (2007).

13. Power, J. D., Mitra, A., Laumann, T. O., Snyder, A. Z., Schlaggar, B. L. & Petersen, S. E. Methods to detect, characterize, and remove motion artifact in resting state fMRI. *Neuroimage* **84**, 320–341 (2014).

14. Abraham, A., Pedregosa, F., Eickenberg, M., Gervais, P., Mueller, A., Kossaifi, J., *et al.* Machine learning for neuroimaging with scikit-learn. *Front. Neuroinform.* **8**, 1–10 (2014).
