# Supplementary material for: Behaviour-correlated profiles of cerebellar-cerebral functional connectivity observed in independent neurodevelopmental disorder cohorts
Source: Transl Psychiatry. 2024 Apr 3;14:173. doi: 10.1038/s41398-024-02857-4 (PMC10991387; doi:10.1038/s41398-024-02857-4)
Supplement: Supplementary file 2 — Supplemental Figures and Tables [file 41398_2024_2857_MOESM2_ESM.docx]

**SUPPLEMENTAL FIGURES & TABLES**


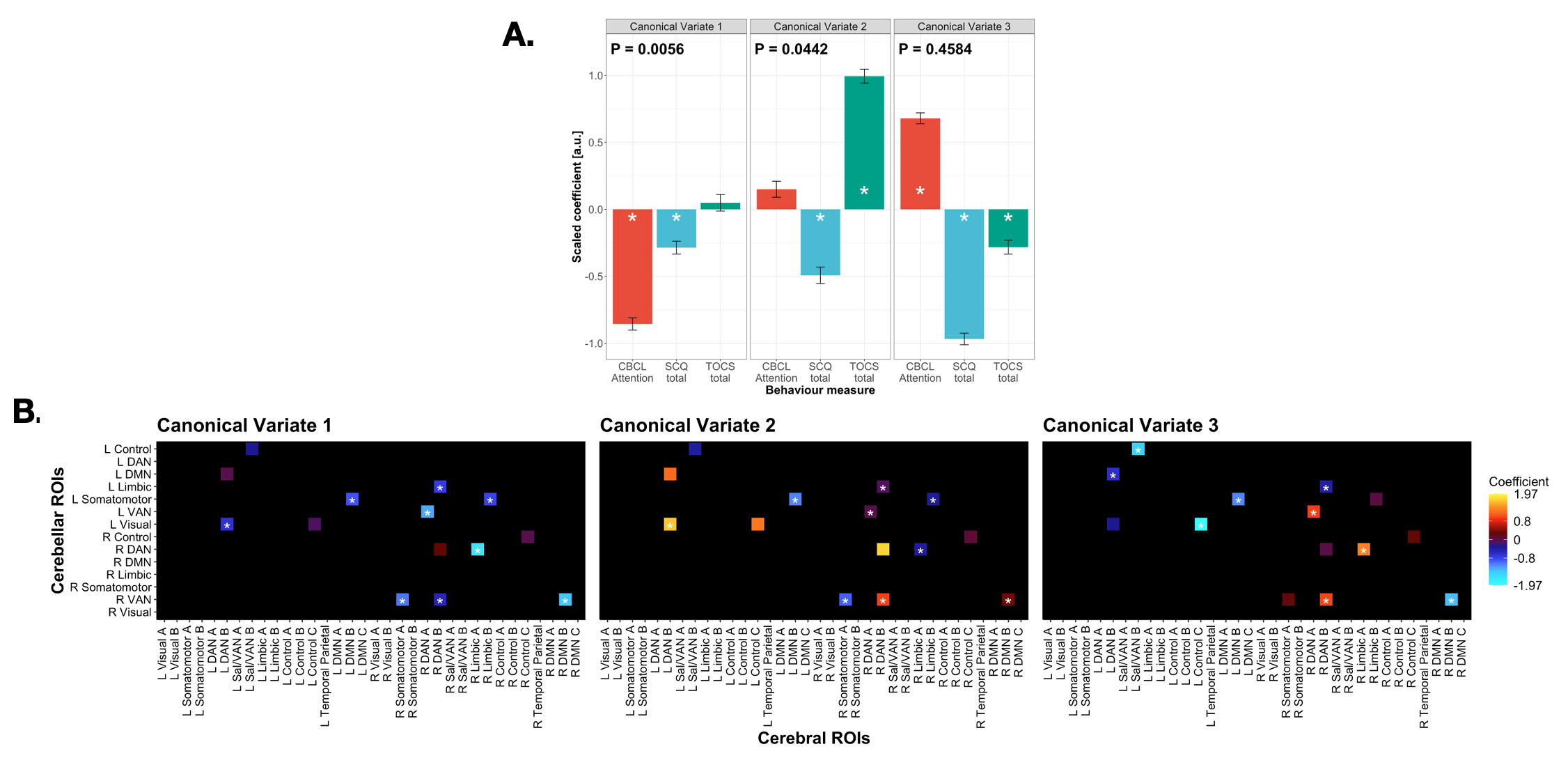


Supplementary Figure 1: (A) Standardized canonical coefficients for behaviour scores measured from the original (i.e., POND) cohort. Only FC features that correlated with behaviour via Spearman’s correlation at a threshold of P < 0.01 were preserved (P < 0.05 was the threshold employed in the main investigation). Errors bars indicate the standard deviation of canonical coefficients recalculated over 10000 bootstrap resamples. * denotes |z| > 1.96, where z is the ratio of a coefficient to its standard error. If |z| > 1.96, the coefficient is considered to be stable. (B) Canonical coefficients for functional connectivity features. * denotes stable coefficients. CBCL = Child Behavior Checklist, SCQ = Social Communication Questionnaire, TOCS = Toronto Obsessive-Compulsive Scale, DAN = dorsal attention network, VAN = ventral attention network, DMN = default mode network, Sal = salience network.


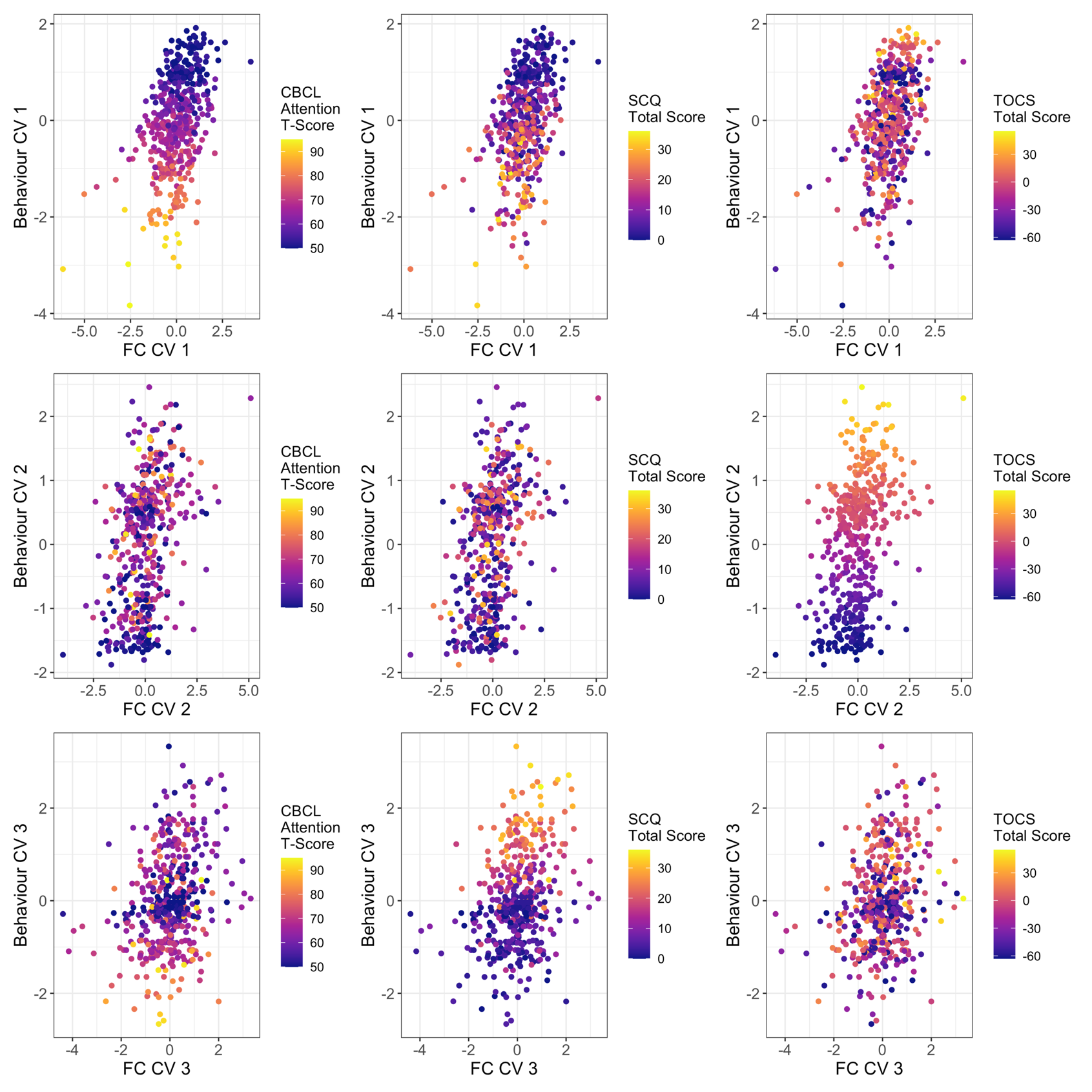


Supplementary Figure 2: Raw behaviour scores as a function of canonical variate loadings for each pair of behaviour and functional connectivity canonical variates for the POND cohort.


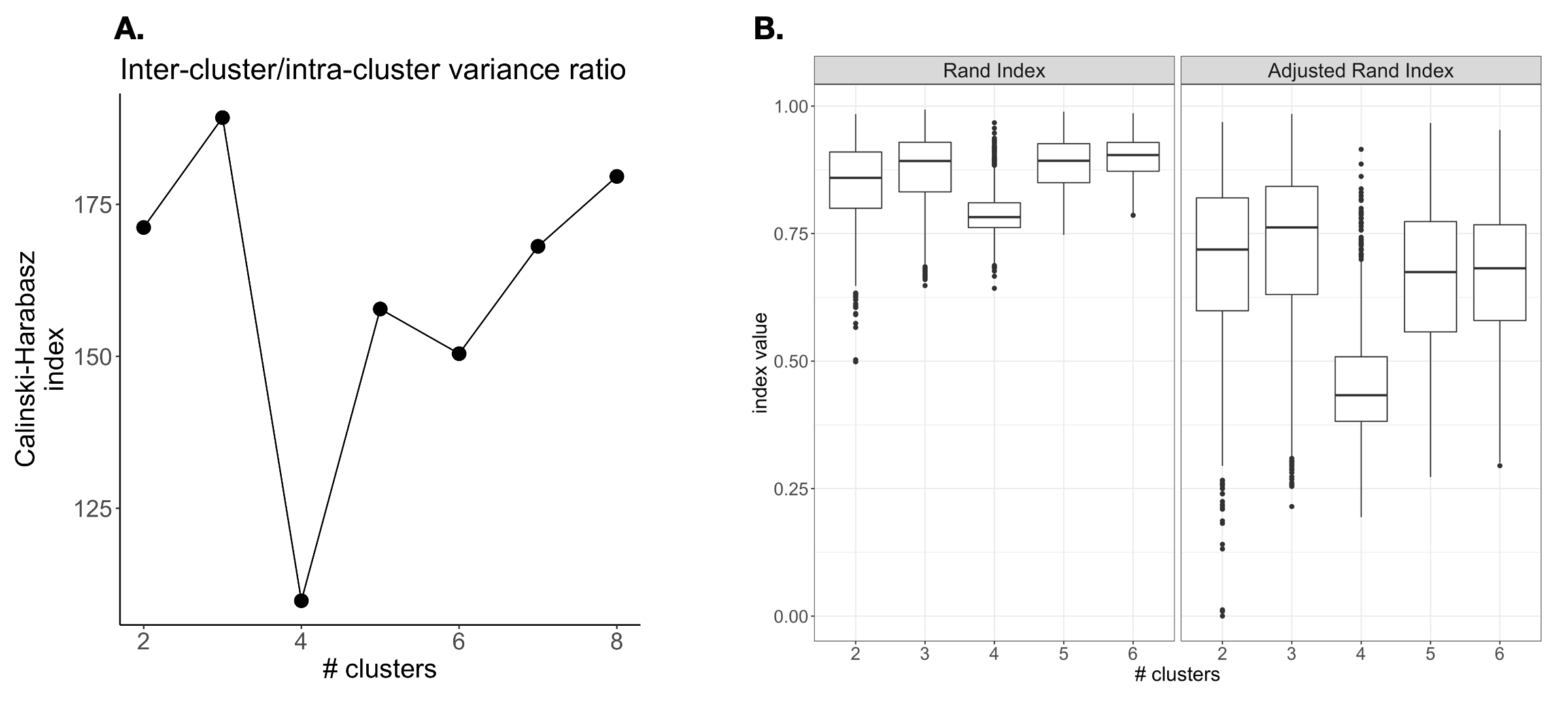


Supplementary Figure 3: (A) Calinhara-Harabasz index and (B) Rand index as a function of number of clusters following spectral clustering of POND functional connectivity loadings from the statistically significant canonical variates. Adjusted Rand index corrects for the expected frequency of co-assignment between two data points for a given number of clusters. Rand index distributions were calculated following bootstrap resampling of subjects 10 000 times.

**IQ**


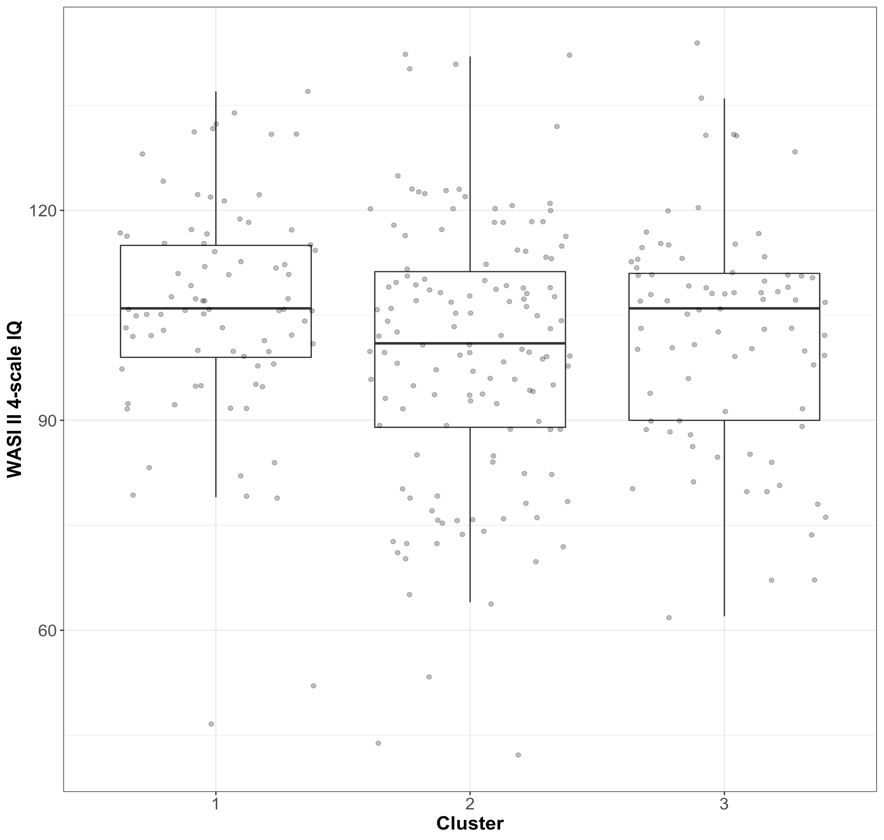


**IQ**

Supplementary Figure 4: IQ as a function of subject cluster for the POND cohort. Only 294 of the 479 subjects had their IQ measured and recorded.


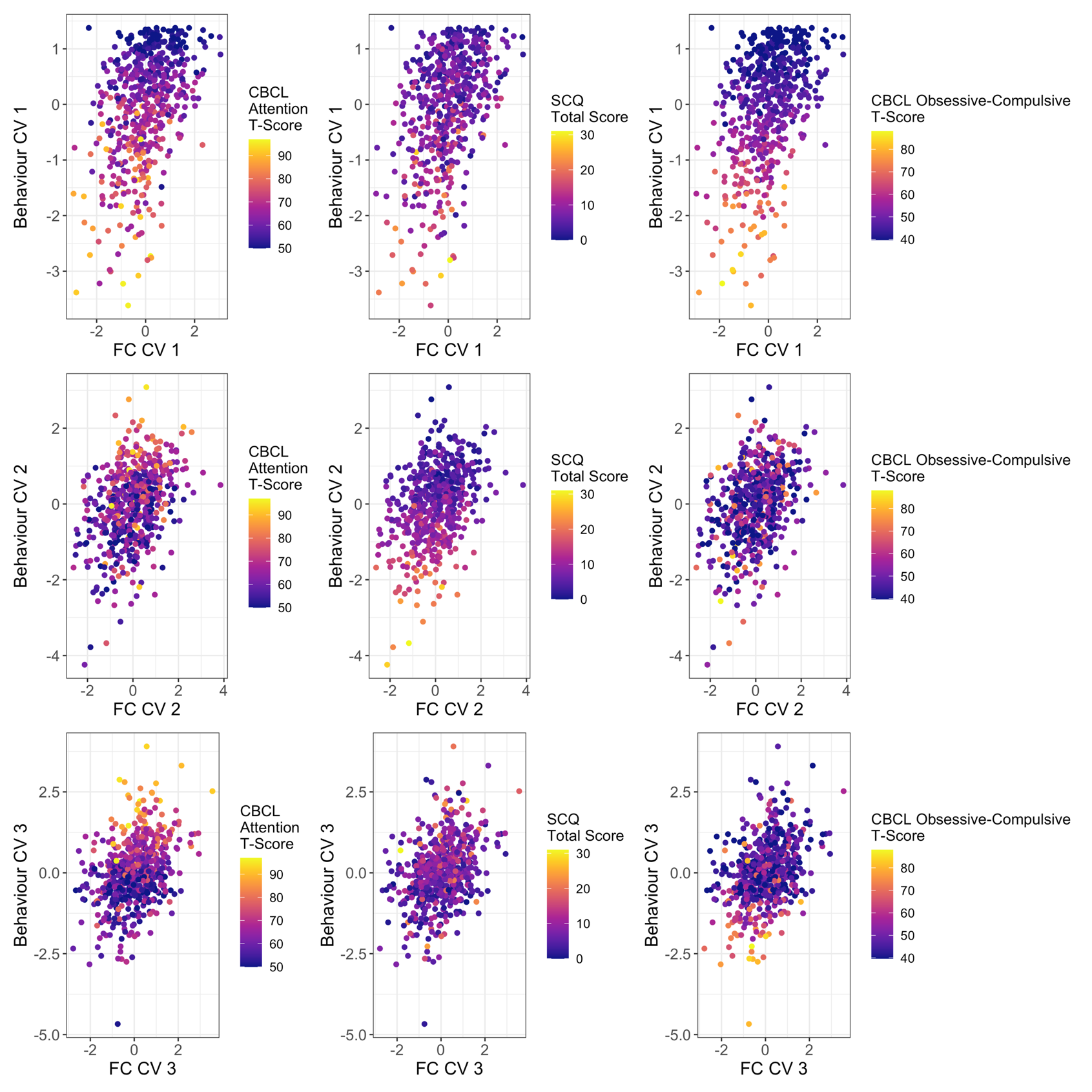


Supplementary Figure 5: Raw behaviour scores as a function of canonical variate loadings for each pair of behaviour and functional connectivity canonical variates for the HBN cohort.


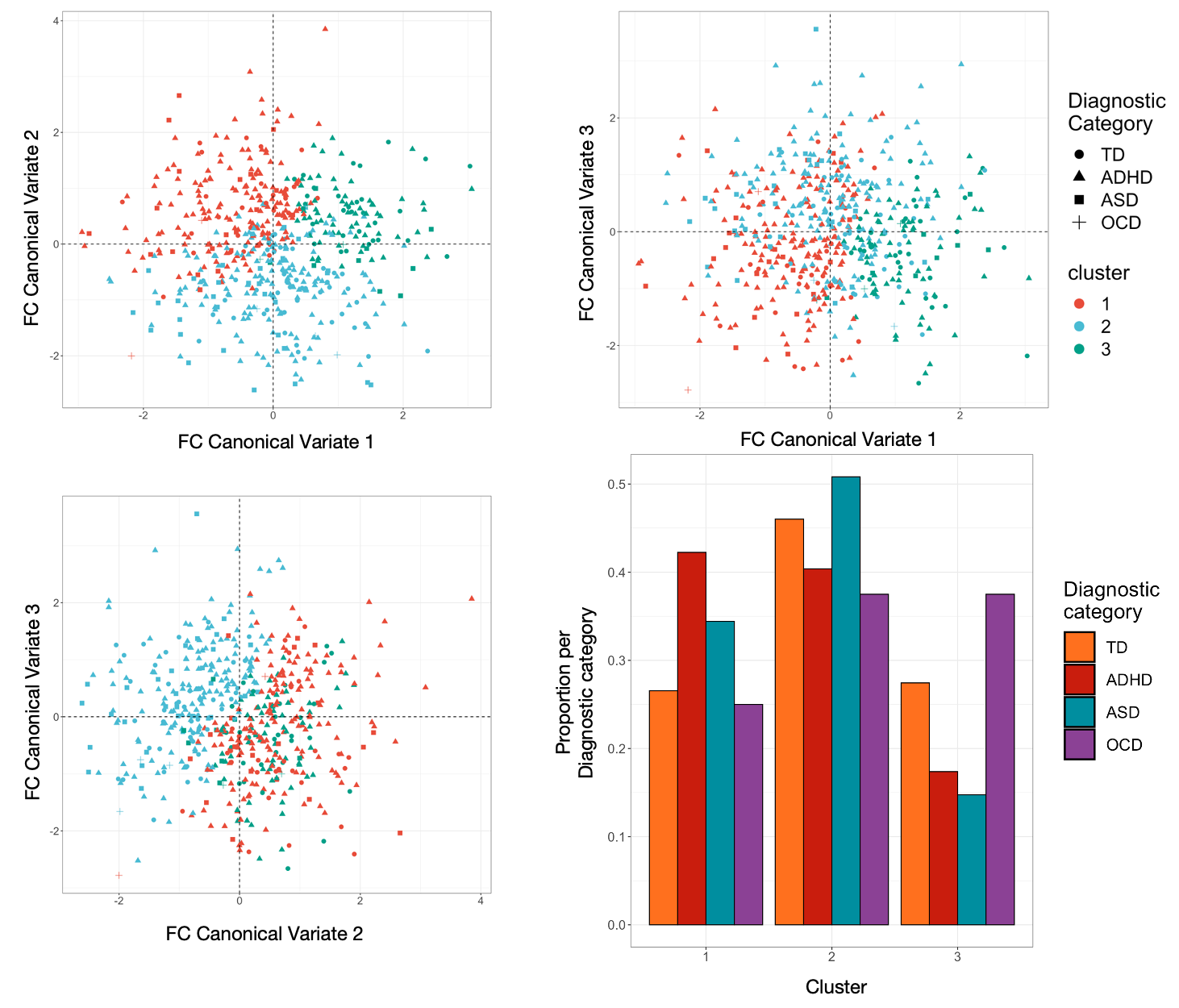


Supplementary Figure 6: Subject cluster assignment following spectral clustering on functional connectivity canonical variate loading values. Bottom Right: Proportion of subjects per diagnostic category per cluster.

|  | **POND** | | | **HBN** | | |
| --- | --- | --- | --- | --- | --- | --- |
|  | **CV 1** | **CV 2** | **CV 3** | **CV 1** | **CV 2** | **CV 3** |
| **Child Behaviour Checklist - Attentional Problems** | -0.82 | 0.16 | -0.72 | -0.39 | 0.59 | 0.89 |
| **Social Communication Questionnaire** | -0.36 | -0.13 | 1.06 | -0.15 | -1.04 | -0.42 |
| **Obsessive-Compulsive Behaviour^i^** | 0.29 | 0.99 | 0.001 | -0.67 | 0.17 | -0.99 |

Supplementary Table 1: Canonical coefficient values for behaviour score canonical variates. Behaviour scores were normalized prior to analysis. Underlined values indicate statistical stability as evaluated by bootstrap resampling ( |coefficient value| / (coefficient standard deviation following 10 000 resamples) > 1.96). i) Scores used to measure obsessive-compulsive behaviour were the Toronto Obsessive-Compulsive Scale total score (POND cohort) and the Child Behaviour Checklist – Obsessive-Compulsive Behaviour subscore (HBN cohort).

| **Functional Connectivity Feature**  **(cerebellum - cerebrum)** | **CV 1** | **CV 2** | **CV 3** | **z (CV 1)** | **z (CV 2)** | **z (CV 3)** |
| --- | --- | --- | --- | --- | --- | --- |
| R DAN - L Visual A | -0.56 | 0.42 | 0.05 | -2.27 | 1.82 | 0.24 |
| L Somatomotor - L Visual B | 0.05 | 0.41 | 0.06 | 0.22 | 1.91 | 0.29 |
| L DMN - L Somatomotor B | 0.33 | 0.54 | 0.74 | 1.26 | 2.02 | 3.02 |
| R DAN - L Somatomotor B | 0.11 | -0.38 | -0.2 | 0.55 | -1.9 | -1.07 |
| R Limbic - L DAN A | -0.51 | 0.58 | 0.08 | -2.34 | 2.84 | 0.38 |
| L Visual - L DAN B | -0.04 | 1.01 | -0.08 | -0.16 | 4.02 | -0.32 |
| L DAN - L DAN B | 0.41 | 0.41 | -0.4 | 1.96 | 1.77 | -1.88 |
| L DMN - L DAN B | -0.17 | 0.71 | 0.13 | -0.71 | 3.06 | 0.57 |
| R Control - L DAN B | 0.7 | 0.21 | 0.82 | 2.6 | 0.72 | 3.05 |
| L Somatomotor - L Sal/VAN A | -0.14 | -0.25 | 0.3 | -0.69 | -1.13 | 1.4 |
| L Control - L Sal/VAN A | 0.31 | 0.24 | -0.39 | 1.55 | 1.14 | -1.9 |
| L Control - L Sal/VAN B | -0.07 | -0.04 | 0.53 | -0.35 | -0.19 | 2.54 |
| R Control - L Sal/VAN B | -0.53 | -0.07 | 0.33 | -2.49 | -0.32 | 1.56 |
| R Control - L Limbic B | -0.36 | 0.18 | -0.27 | -1.91 | 0.9 | -1.44 |
| L DAN - L Limbic A | -0.11 | 0.59 | -0.2 | -0.49 | 2.54 | -0.9 |
| R Visual - L Limbic A | -0.36 | -0.03 | 0.04 | -1.81 | -0.12 | 0.19 |
| L Visual - L Control C | -0.34 | 0.71 | 0.59 | -1.19 | 2.65 | 2.2 |
| L Control - L Control C | -0.44 | 0.36 | 0.37 | -1.91 | 1.52 | 1.61 |
| R Visual - L Control C | -0.9 | 0.12 | 0.74 | -3.58 | 0.45 | 3.14 |
| R Somatomotor - L Control A | -0.05 | 0.26 | 0.35 | -0.23 | 1.17 | 1.66 |
| R Control - L Control A | 0.62 | 0.25 | 0.12 | 3.14 | 1.16 | 0.59 |
| L VAN - L Control B | -0.42 | -0.02 | -0.77 | -1.98 | -0.08 | -3.69 |
| R DAN - L Control B | -1.06 | -0.61 | -0.2 | -4 | -1.98 | -0.76 |
| R VAN - L Temporal Parietal | 0.01 | 0.37 | 0.23 | 0.05 | 1.79 | 1.13 |
| R DMN - L Temporal Parietal | -0.42 | 0.25 | 0.27 | -1.86 | 1.12 | 1.25 |
| L Control - L DMN C | -0.04 | 0.25 | -0.11 | -0.22 | 1.32 | -0.57 |
| R Visual - L DMN C | -0.19 | 0.67 | 0.14 | -0.75 | 2.7 | 0.58 |
| R DAN - L DMN C | 0.11 | 0.76 | 0.26 | 0.46 | 3.3 | 1.18 |
| R Limbic - L DMN C | -0.54 | 0.26 | 0.15 | -2.35 | 1.14 | 0.7 |
| L DAN - L DMN A | 0.31 | 0.99 | 0.15 | 1.14 | 3.48 | 0.57 |
| R Somatomotor - L DMN A | -0.32 | 0.72 | 0.24 | -1.23 | 3.03 | 1 |
| L Somatomotor - L DMN B | -0.51 | 0.13 | 0.82 | -2.31 | 0.6 | 4.13 |
| L Control - L DMN B | 0.06 | 0.31 | 0.35 | 0.25 | 1.39 | 1.64 |
| R Control - R Visual A | -0.44 | -0.22 | 0.82 | -2.01 | -0.93 | 3.98 |
| R Visual - R Visual B | 0.12 | 0.27 | -0.22 | 0.6 | 1.36 | -1.13 |
| L Visual - R Somatomotor A | -0.16 | 0.14 | 0.58 | -0.8 | 0.65 | 2.95 |
| L Control - R Somatomotor A | -0.11 | 0.6 | 0.52 | -0.5 | 2.76 | 2.47 |
| R VAN - R Somatomotor A | -0.63 | -0.29 | -0.11 | -2.9 | -1.27 | -0.5 |
| L VAN - R DAN A | -0.75 | 0.27 | -0.54 | -2.92 | 1.07 | -2.3 |
| L VAN - R DAN B | 0.3 | 0.96 | -0.33 | 1.29 | 3.97 | -1.41 |
| L Limbic - R DAN B | -0.16 | 0.18 | 0.61 | -0.81 | 0.87 | 3.23 |
| R DAN - R DAN B | 0.18 | 0.73 | -0.21 | 0.73 | 2.82 | -0.86 |
| R VAN - R DAN B | -0.55 | 0.72 | -0.75 | -2.05 | 2.75 | -3.02 |
| R DMN - R DAN B | -0.24 | 0.22 | -0.26 | -1.01 | 0.93 | -1.13 |
| L Somatomotor - R Sal/VAN A | 0 | -0.27 | 0.65 | 0.01 | -1.19 | 3.21 |
| L DAN - R Sal/VAN B | 0.03 | 0.38 | -0.79 | 0.13 | 1.6 | -3.25 |
| L DMN - R Sal/VAN B | -0.18 | -0.49 | 0.95 | -0.7 | -1.81 | 3.89 |
| R Visual - R Sal/VAN B | 0.34 | 0.54 | 1.3 | 1.26 | 1.8 | 4.94 |
| R Somatomotor - R Sal/VAN B | -0.83 | -0.16 | 0.57 | -3.29 | -0.57 | 2.3 |
| L Somatomotor - R Limbic B | -0.5 | -0.18 | -0.39 | -2.36 | -0.81 | -1.87 |
| L Control - R Limbic B | -0.98 | -0.05 | 0.34 | -3.95 | -0.18 | 1.46 |
| R DAN - R Limbic A | -0.9 | -0.14 | -0.3 | -3.69 | -0.54 | -1.31 |
| R Limbic - R Control C | -0.39 | 0.88 | -0.22 | -1.4 | 3.45 | -0.85 |
| R Control - R Control C | -0.78 | -0.04 | -0.42 | -3.23 | -0.15 | -1.81 |
| L Somatomotor - R Control A | 0.47 | 0.23 | -0.07 | 2.06 | 0.92 | -0.3 |
| L DMN - R Temporal Parietal | -0.16 | 0.36 | -0.3 | -0.76 | 1.88 | -1.52 |
| L Visual - R DMN C | -0.38 | -0.51 | -0.54 | -1.69 | -2.17 | -2.52 |
| L DMN - R DMN A | -0.56 | -0.19 | -0.24 | -2.62 | -0.81 | -1.07 |
| L Limbic - R DMN B | 0.19 | 0.37 | 0.66 | 0.8 | 1.58 | 3.03 |
| R VAN - R DMN B | -0.95 | 0.35 | 0.71 | -3.16 | 1.16 | 2.77 |
| R Control - R DMN B | 0.36 | -0.68 | 1.61 | 1.27 | -2.13 | 5.88 |

Supplementary Table 2: Canonical coefficients and z-scores for functional connectivity canonical variates in the POND cohort.

|  | **POND (Original Cohort)** | | | | **HBN (Replication Cohort)** | | | |
| --- | --- | --- | --- | --- | --- | --- | --- | --- |
|  | **F** | **df 1** | **df 2** | **P-value** | **F** | **df 1** | **df 2** | **P-value** |
| **Framewise Displacement** | 0.19 | 2 | 475 | 0.83 | 0.37 | 2 | 553 | 0.69 |
| **Age** | 0.13 | 2 | 475 | 0.88 | 0.05 | 2 | 553 | 0.95 |
| **IQ** | 3.53 | 2 | 291 | 0.03 | 0.69 | 2 | 480 | 0.50 |

Supplementary Table 3: ANOVA results for assessing difference in in-scanner motion (measured as framewise displacement), age, and IQ) between subject clusters for the POND cohort.

| **Functional Connectivity Feature**  **(cerebellum – cerebrum)** | **CV 1** | **CV 2** | **CV 3** | **z (CV 1)** | **z (CV 2)** | **z (CV 3)** |
| --- | --- | --- | --- | --- | --- | --- |
| L Visual - L Visual A | -2.32 | -0.31 | 1.37 | -2.48 | -0.31 | 1.48 |
| L VAN - L Visual A | -1.17 | 1.87 | 2.44 | -1.5 | 2.46 | 3.46 |
| R Somatomotor - L Visual A | -2.56 | 1.16 | 0.65 | -3.06 | 1.42 | 0.84 |
| L Control - L Visual B | -1.59 | 0.95 | 0.16 | -2.17 | 1.3 | 0.23 |
| L Visual - L Somatomotor A | -1.03 | -3.97 | -1 | -0.96 | -3.73 | -0.96 |
| R Control - L DAN A | -1.85 | 0.64 | -0.21 | -2.4 | 0.85 | -0.3 |
| R DMN - L DAN A | -0.05 | -2.62 | -0.23 | -0.07 | -3.63 | -0.32 |
| L DMN - L DAN B | -0.35 | 1.43 | 0.09 | -0.52 | 2.07 | 0.12 |
| L Control - L Sal/VAN B | -1.73 | -1.84 | 0.41 | -2.2 | -2.28 | 0.55 |
| L DAN - L Limbic B | -1.37 | -0.27 | 1.17 | -2.08 | -0.4 | 1.84 |
| R DAN - L Limbic B | -0.49 | -1.21 | -0.39 | -0.74 | -1.72 | -0.58 |
| L Visual - L Limbic A | -0.94 | -4.26 | 4.47 | -0.74 | -3.3 | 3.78 |
| L Somatomotor - L Limbic A | -1.73 | -0.4 | 3.11 | -1.98 | -0.42 | 3.69 |
| R Visual - L Limbic A | -0.67 | -1.07 | 3.06 | -0.69 | -1.1 | 3.45 |
| L VAN - L Control C | -2.68 | -2.72 | -0.6 | -2.85 | -2.72 | -0.67 |
| R VAN - L Control C | -3.06 | 3.18 | -2.6 | -2.9 | 3.05 | -2.68 |
| R DMN - L Control C | -0.84 | 0.46 | 1.87 | -1.3 | 0.7 | 3.04 |
| L Control - L Temporal Parietal | -0.36 | -0.82 | -3 | -0.5 | -1.05 | -4.43 |
| L Visual - L DMN C | -1.5 | 1.05 | 3.16 | -1.53 | 1.15 | 3.65 |
| L VAN - L DMN C | -1.23 | -0.87 | 2.13 | -1.57 | -1.12 | 2.96 |
| R Visual - L DMN A | 0.94 | -1.86 | -1.43 | 1.09 | -2.21 | -1.76 |
| R DAN - L DMN A | -1.23 | 0.92 | 3.7 | -1.39 | 1.05 | 4.85 |
| L DAN - L DMN B | -0.44 | -2.99 | 1.36 | -0.53 | -3.7 | 1.74 |
| L VAN - L DMN B | -1.26 | 0.12 | 0.82 | -1.83 | 0.17 | 1.25 |
| R Visual - R Visual A | -1.05 | 0.61 | 0.94 | -1.37 | 0.81 | 1.3 |
| L DAN - R Visual B | 0.29 | 1.83 | 2.66 | 0.34 | 2.13 | 3.48 |
| L Limbic - R Visual B | -0.04 | -2.51 | 1.18 | -0.04 | -3.15 | 1.52 |
| R VAN - R Visual B | -1.49 | 1.33 | -3.74 | -1.62 | 1.32 | -4.3 |
| L Control - R Somatomotor A | -2.84 | 0.95 | -1.05 | -3.44 | 1.12 | -1.34 |
| R Limbic - R Somatomotor A | -3.23 | -0.61 | -0.62 | -3.61 | -0.63 | -0.7 |
| R Somatomotor - R Somatomotor B | 1.16 | -3.73 | -0.27 | 1.2 | -4.14 | -0.3 |
| R DAN - R Somatomotor B | -1.53 | 0.87 | 2.69 | -1.76 | 1 | 3.36 |
| L DAN - R DAN B | -2.49 | 1.38 | 0.37 | -3.01 | 1.67 | 0.49 |
| R Control - R DAN B | -3.03 | -0.6 | 1.75 | -3.46 | -0.64 | 2.12 |
| L Visual - R Sal/VAN A | -3.28 | -2.88 | 1.02 | -3.14 | -2.54 | 0.98 |
| L Somatomotor - R Sal/VAN A | -1.17 | -0.16 | -0.87 | -1.41 | -0.2 | -1.16 |
| L Control - R Sal/VAN A | -2.52 | 0.51 | -2.54 | -2.94 | 0.56 | -3.05 |
| L VAN - R Sal/VAN B | 1.29 | -2.16 | 0.58 | 1.72 | -2.98 | 0.84 |
| R DMN - R Limbic B | -1.56 | 1.19 | 1.66 | -2.11 | 1.72 | 2.59 |
| R Limbic - R Limbic A | -3.87 | -1.21 | -1.79 | -4.15 | -1.14 | -1.95 |
| L Visual - R Control C | -0.35 | -1.74 | -3.25 | -0.38 | -1.82 | -3.8 |
| L Somatomotor - R Control C | -1.85 | 3.63 | 1.64 | -1.71 | 3.59 | 1.69 |
| R Somatomotor - R Control B | -1.43 | -0.02 | 2.32 | -1.83 | -0.02 | 3.16 |
| R VAN - R Control B | -3.92 | -1.42 | 2 | -4.08 | -1.37 | 2.17 |
| L Limbic - R Temporal Parietal | 1.58 | -4.48 | 0.29 | 1.42 | -4.4 | 0.28 |
| R DAN - R Temporal Parietal | -1.89 | 2.48 | -2.55 | -2.06 | 2.67 | -3.01 |
| R DMN - R Temporal Parietal | -0.71 | -1.34 | 0.95 | -1.07 | -1.99 | 1.52 |
| R Visual - R DMN C | -1.72 | 0.19 | -3.14 | -1.85 | 0.19 | -3.61 |
| R VAN - R DMN C | -3.42 | -0.52 | -1.75 | -3.69 | -0.52 | -1.99 |
| R Visual - R DMN B | -3.77 | 1.94 | -3.07 | -3.39 | 1.65 | -2.93 |
| R Control - R DMN B | -1.91 | -2.93 | 0.32 | -2.32 | -3.36 | 0.38 |

Supplementary Table 4: Canonical coefficients and z-scores for functional connectivity canonical variates in the HBN cohort.

|  | **POND CV 1** | **POND CV 2** | **POND CV 3** | **Common FC features independent of stability** |
| --- | --- | --- | --- | --- |
| **HBN CV 1** | Control - DAN  Control - Sal/VAN  VAN - Control  Control - Visual  Somatomotor - Limbic  DAN - Limbic  VAN - DMN | DAN - Limbic  Visual - DMN  Control - Somatomotor  DAN - DAN  Control - DMN | Control - DAN  Control - Sal/VAN  VAN - Control  Control - Visual  Control - Somatomotor  Visual - Sal/VAN  Visual - DMN  VAN - DMN  Control - DMN | DAN - Visual  Somatomotor - Visual  DAN - Somatomotor  DAN - DAN  DMN - DAN  Control - DAN  Somatomotor - Sal/VAN  Control - Sal/VAN  DAN - Limbic  Visual - Limbic  Visual - Control  Somatomotor - Control  VAN - Control  DMN – Temporal Parietal  Control - DMN  Visual - DMN  DAN - DMN  Control - Visual  Visual -Visual  Visual - Somatomotor  Control - Somatomotor  Visual - Sal/VAN  Somatomotor - Limbic  VAN - DMN |
| **HBN CV 2** | DAN-Visual  Control - Sal/VAN  VAN - Control  Somatomotor - Control | DMN - DAN  Visual - DMN  DAN - DMN  Control - DMN | Control - Sal/VAN  VAN - Control  Visual - Somatomotor  Visual - Sal/VAN  Visual - DMN  Control - DMN |  |
| **HBN CV 3** | DAN - Visual  Control - DAN  Control - Sal/VAN  Visual - Control  VAN - Control  Somatomotor - Limbic  Somatomotor - Control  VAN - DMN | Visual - Control  Visual - DMN  DAN - DMN | Control - DAN  Control - Sal/VAN  Visual - Control  VAN - Control  Visual - DMN  VAN - DMN |  |

Supplementary Table 5: Shared stable functional connectivity features between POND (original cohort) and HBN (replication cohort) canonical variates. Canonical variates were compared over a coarser parcellation in which subdivisions of the same network (e.g. DMN A and DMN B) were combined into a single bilateral region.
